# mSphere of Influence: Tweaking Organellar Purification Approaches

**DOI:** 10.1128/mSphere.00690-20

**Published:** 2020-09-09

**Authors:** Diego Huet

**Affiliations:** a Department of Pharmaceutical and Biological Sciences, University of Georgia, Athens, Georgia, USA; b Center for Tropical and Emerging Global Diseases, University of Georgia, Athens, Georgia, USA

**Keywords:** apicomplexans, mitochondria, organellar isolation

## Abstract

Diego Huet studies the organelles involved in the metabolic adaptations of the apicomplexan parasite Toxoplasma gondii. In this mSphere of Influence article, he reflects on how the paper “Absolute quantification of matrix metabolites reveals the dynamics of mitochondrial metabolism” by Chen et al. (W. W. Chen, E. Freinkman, T. Wang, K. Birsoy, and D. M. Sabatini, Cell 166:1324–1337.e11, 2016, https://doi.org/10.1016/j.cell.2016.07.040) shaped his research by providing an approach for rapidly and specifically isolating mitochondria to probe the metabolism of these organelles.

## COMMENTARY

Every organism needs to adapt to the ever-changing conditions that it encounters in its environment. Cells must regulate their metabolism depending on nutrient availability, and mitochondria play a central role in this process. Cellular homeostasis is achieved as metabolites are exported and imported to mitochondria, and scientists have tried to capture a glimpse of those metabolic fluxes using different approaches. However, the majority of the existing methods involve long ultracentrifugation steps, resulting in a significant loss of mitochondrial metabolites that also often yield purified fractions with unwanted cytoplasmic material and other organelles. To overcome those hurdles, the Sabatini group (1) developed a new methodology that allows for the rapid immunocapture of epitope-tagged mitochondria followed by metabolite profiling by liquid chromatography and mass spectrometry (LC/MS). The approach allowed the authors to assess the mitochondrial metabolome, the MITObolome, and demonstrated the dynamics of mitochondrial metabolism after respiratory chain dysfunction.

To isolate mitochondria in a rapid and specific manner, the Sabatini group relied on an immunopurification approach that uses outer mitochondrial proteins as handles for immunocapturing the organelle. Using an epitope-tagged recombinant protein that they named MITO-tag, the group successfully isolated mitochondria after only 3.5 min of immunocapture. The mitochondria isolated by this approach showed good purity and integrity, and the authors were able to quantify the concentrations of a list of 132 predicted mitochondrial metabolites in the MITObolome by LC/MS. From homogenization to metabolite extraction, the procedure took a total of 12 min, an incredibly short time compared to other approaches used to isolate mitochondria. The purification approach also allowed them to determine how the mitochondrial metabolome changed after treating the cells with different respiratory chain inhibitors. Interestingly, analysis of the mitochondrial metabolome after these treatments revealed that each inhibitor yielded a unique mitochondrial metabolic signature. For example, the results show that inhibition of the respiratory chain triggers an accumulation of aspartate in the mitochondrial matrix, likely due to the inhibition of mitochondrial glutamate dehydrogenases.

My laboratory studies apicomplexans, a group of single-celled eukaryotic parasites. We particularly focus on Toxoplasma gondii, the causative agent of toxoplasmosis. This parasite has a wide array of hosts—virtually almost any warm-blooded animal—but its sexual cycle takes place only in the cells of the small intestine of felids. The parasite has a single, large mitochondrion, and during its life cycle it alternates between a fast-replicating form and a slow-replicating, cyst-forming stage. My laboratory aims at understanding how T. gondii uses its mitochondrion to adapt to the different environments it can encounter during its life cycle. One of the approaches we are developing consists of isolating the mitochondrion from different forms of the parasite to assess their metabolic activity. The paper from the Sabatini group offered us an interesting methodology and workflow that we are currently trying to adapt to T. gondii. As is common when dealing with unconventional model organisms, adapting a technique established in a mammalian system can be difficult and often requires significant optimization steps. My group is currently tweaking the MITO-tag approach from the Sabatini group to rapidly isolate the mitochondrion of T. gondii. Achieving a fast mitochondrial isolation would then allow us to assess the MITObolome of parasites exposed to different environments or sampled from different life stages in an unprecedented way, contributing to the understanding of apicomplexan metabolism. As someone studying T. gondii, these techniques could offer a way to isolate not only the mitochondrion of the parasite but also other organelles such as the apicoplast, a plant-like plastid involved in many metabolic pathways.

Since the publication of this method (1), the Sabatini group has expanded this approach to other organelles. The group was able to rapidly isolate lysosomes (2) and peroxisomes (3). Former members of the Sabatini group also generated a MITO-tag mouse, allowing for the specific isolation of mouse hepatocyte mitochondria *in vivo* to assess their metabolome (4). As the techniques and workflows for organellar isolation continue to evolve, other approaches have been recently developed. One of the most recent purification methods, which successfully isolated mitochondria, lysosomes, and peroxisomes, relies on the interaction between the twin strep tag and streptavidin variants (5). In theory, all of these organellar isolation techniques could be adapted to any organism, including T. gondii, provided that the molecular handle used for the immunoprecipitation is targeted to the desired organelle. It is all a matter of tweaking the techniques to your favorite organism.
